# Enhanced Antifungal Activity by Ab-Modified Amphotericin B-Loaded Nanoparticles Using a pH-Responsive Block Copolymer

**DOI:** 10.1186/s11671-015-0969-1

**Published:** 2015-06-10

**Authors:** Xiaolong Tang, Jingjing Dai, Jun Xie, Yongqiang Zhu, Ming Zhu, Zhi Wang, Chunmei Xie, Aixia Yao, Tingting Liu, Xiaoyu Wang, Li Chen, Qinglin Jiang, Shulei Wang, Yong Liang, Congjing Xu

**Affiliations:** Stem Cell Engineering Research Center, Anhui University of Science and Technology, Huainan, 232001 China; Department of Respiration, Tumour Hospital of Affiliated Huainan Oriental Hospital Group, Anhui University of Science and Technology, Huainan, 232035 China; School of Biotechnology, Southern Medical University, Guangzhou, 510515 China; Yantai City Center for Disease Control and Prevention, Yantai, 264003 China; Clinical Laboratory, Department of Nephrology, Huai’an Hospital Affiliated of Xuzhou Medical College, Huaian, 223002 China

**Keywords:** Amphotericin B, Targetability, *C. albicans*, Nanocarrier

## Abstract

Fungal infections are an important cause of morbidity and mortality in immunocompromised patients. Amphotericin B (AMB), with broad-spectrum antifungal activity, has long been recognized as a powerful fungicidal drug, but its clinical toxicities mainly nephrotoxicity and poor solubility limit its wide application in clinical practice. The fungal metabolism along with the host immune response usually generates acidity at sites of infection, resulting in loss of AMB activity in a pH-dependent manner. Herein, we developed pH-responsive AMB-loaded and surface charge-switching poly(d,l-lactic-*co*-glycolic acid)-*b*-poly(l-histidine)-*b*-poly(ethylene glycol) (PLGA-PLH-PEG) nanoparticles for resolving the localized acidity problem and enhance the antifungal efficacy of AMB. Moreover, we modified AMB-encapsulated PLGA-PLH-PEG nanoparticles with anti-*Candida albicans* antibody (CDA) (CDA-AMB-NPs) to increase the targetability. Then, CDA-AMB-NPs were characterized in terms of physical characteristics, in vitro drug release, stability, drug encapsulation efficiency, and toxicity. Finally, the targetability and antifungal activity of CDA-AMB-NPs were investigated in vitro*/*in vivo. The result demonstrated that CDA-AMB-NPs significantly improve the targetability and bioavailability of AMB and thus improve its antifungal activity and reduce its toxicity. These NPs may become a good drug carrier for antifungal treatment.

## Background

Amphotericin B (AMB), which has been considered to associate with ergosterol in fungal cell membranes to mediate ion-permeable pores, is still one of the most effective agents for the treatment against systemic fungal infections [1–3]. However, the association leading to the elevation of the intracellular Ca^2+^ concentration can result in renal tubular cell injury [3]. Recently, new lipid formulations of AMB have been developed to reduce the nephrotoxicity and improve the therapeutic index [4, 5], while new lipid formulations still remain limited to be used mainly due to the high cost.

In recent years, advances in gaining drug potency or in vivo properties of nanoparticles (NPs) for drug delivery applications by increasing targetability and environmental sensing leading to NP property switching and sustained drug release have been improving the bioavailability of AMB with few side effects for the treatment against systemic fungal infections. The fungal metabolism and the host immune response often cause localized acidity, which results loss of drug activity in a pH-dependent manner [6, 7]. Fungi can adjust to local conditions such as ambient pH, temperature, and nutrient supply through shared metabolic systems including the Pal/PacC pathway in *Aspergillus* sp*.* and the RIM101 pathway in *Candida* sp*.* to infect tissue [8–10].

Developing systemically available nanodrug carriers that can target and improve antibiotic properties in the setting of localized acidity may therefore be a method to improve the treatment of these and potentially other infections [9, 10]. Herein, we developed pH-responsive, drug-encapsulated, surface charge-switching poly(d,l-lactic-*co*-glycolic acid)-*b*-poly(l-histidine)-*b*-poly(ethylene glycol) (PLGA-PLH-PEG) nanoparticles for treating fungal infections. The PLH segment would become positively charged under acidic conditions, imparting the PLGA-PLH-PEG NP surface an overall positive zeta potential, facilitating interactions with the negatively charged elements of the fungal cell wall leading to strong multivalent electrostatic-mediated binding. At the same time, in order to further increase the targeting efficacy for fungal infections, we prepared anti-*Candida albicans* antibody (CDA)-modified AMB-loaded PLGA-PLH-PEG nanoparticles (CDA-AMB-NPs). As CDA reacts with antigens presenting at the cell wall of *C. albicans*, these CDA-AMB-NPs delivering AMB efficiently are designed to target to and bind avidly to *C. albicans* in acidity [11, 12], reducing the loss of antifungal activity in acidity.

The result demonstrates that CDA-AMB-NPs switched their surface charge from a negative zeta potential (−3.82 ± 0.53 mV) at pH 7.35 to a positive one (2.81 ± 0.34 mV) at pH 6.8, yielding a significant increase in targetability and antifungal efficacy at pH 6.8 compared to pH 7.35 with reduced hemolytic activity and cytotoxicity on human renal tubular epithelial cells.

Herein, the CDA-AMB-NPs might step up the development of systemically administered and targeted drug carriers that can potentially treat systemic candidiasis infection associated with acidity.

## Materials and Methods

### Materials

All chemical reagents used in this study were of HPLC grade and were purchased from Sigma-Aldrich, St. Louis, MO, USA, unless otherwise noted. l-Histidine (His), AMB, and coumarin 6 were purchased from Sigma-Aldrich (St. Louis, MO, USA). Thionyl chloride and PLGA-*N*-hydroxysuccinimide (NHS) were obtained from the Graduate School at Shenzhen, Tsinghua University. l-His was derivatized by introducing a carbobenzoxy (CBZ) group to the α-amino group; the amino group in the imidazole ring of *N*_α_-CBZ-l-His was protected with a dinitrophenyl (DNP) group. *N*_α_-CBZ-*N*^*im*^-DNP-l-His was then transformed to *N*-carboxy anhydride (NCA) form by thionyl chloride. Acetonitrile and methanol were purchased from EM Science (Chrom AR, HPLC grade, Mallinckrodt Baker, USA). All the other chemicals and reagents used were of analytical grade and commercially available. Millipore water was prepared by the Milli-Q Plus System (Millipore Corporation, Bedford, MA, USA). *C. albicans* (ATCC 90029) were obtained from the American Type Culture Collection (ATCC; Rockville, MD, USA). mPEG-OPSS polymer (MW 5000) and mPEG-NH2 (MW 5000) were obtained from Laysan Bio (Arab, AL, USA). Phosphate-buffered solution (PBS) and fetal bovine serum (FBS) were purchased from Gibco Co. (Uxbridge, UK).

### Polymer Synthesis

**Fig. 1:**
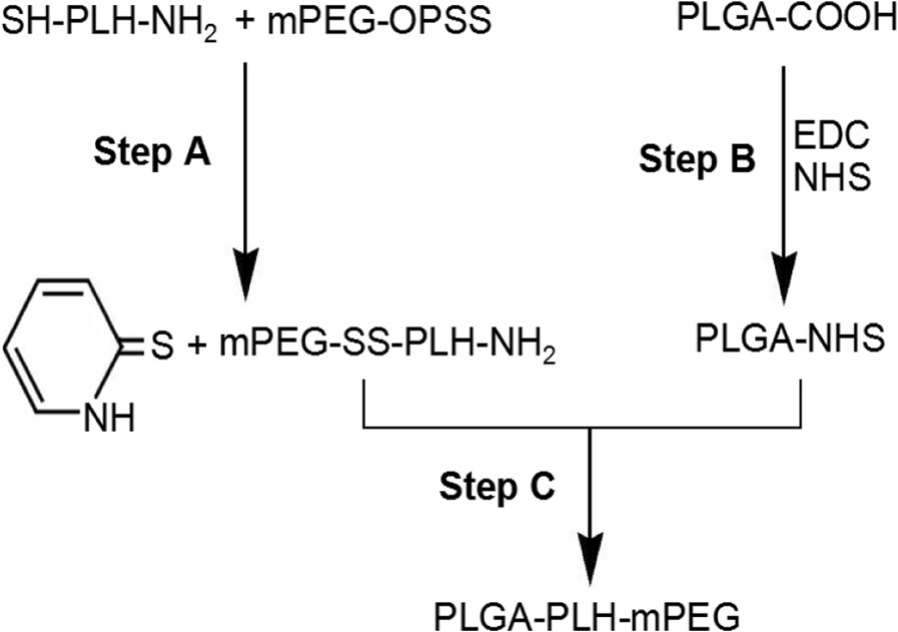
Synthesis procedure for forming the PLGA-PLH-PEG copolymer. End-grafting strategy was employed to form the desired PLGA-PLH-PEG copolymer. The C-terminal Cys of the PLH block reacts with the orthopyridyldisulfide-modified end group of PEG to form the PLH-PEG copolymer and then activates the PLGA-COOH using 1-ethyl-3-(3-dimethylaminopropyl) carbodiimide (EDC) and *N*-hydroxysuccinimide (NHS). Lastly, PEG-PLH and PLGA-NHS polymers are conjugated using the N-terminal Lys to form the desired PLGA-PLH-PEG copolymer

Poly(d,l-lactide-*co*-glycolide)-*b*-poly(l-histidine)-*b*-poly(ethylene glycol) (PLGA-PLH-PEG) was synthesized by a sequential end-grafting process. First, for poly(l-histidine)-*b*-poly(ethylene glycol) (PLH-PEG) synthesis, NCA was synthesized by thionyl chloride treatment of *N*^α^-CBZ-*N*^*im*^-DNP-l-His. Then, ring-opening polymerization of the NCA was initiated by isopropylamine to produce the product poly(*N*^*im*^-DNP-l-His), which was further coupled with carboxylated PEG to produce NH_2_-PLH-PEG diblock copolymer under thiolysis deprotection using 2-mercaptoethanol [13]. Next, PLGA-NHS and PLH-PEG were reacted with *N*,*N*-diisopropylethylamine (DIEA) for 24 h at 20 °C. The resulting crude triblock copolymer PLGA-PLH-PEG was formulated (Fig. 1).

### Fabrication of CDA-AMB-NPs and Characterization

Modified emulsion/solvent evaporation techniques were utilized to form AMB-loaded NPs [14]. In brief, a pre-weighed amount of AMB powder and 10.0 mg of PLGA-PLH-PEG copolymer were dissolved in a volume of 5.0 mL of 15:85 *v*/*v* DMSO/ethyl acetate solution by vortexing and sonication. The resulted nanoparticle suspension was stirred at room temperature overnight and centrifuged at 25,000 rpm for 20 min. The AMB-loaded NPs were then hardened by allowing slow organic solvent evaporation for 24 h in the hood. AMB-loaded NPs were purified by triple filtration using Amicon Ultra-4 100,000 NMWL centrifugal filter units (Millipore, Billerica, MA, USA) using sterile water and freeze-dried using 10 % *w*/*v* of sucrose as a cryoprotectant.

To form CDA-modified NPs, AMB-loaded PLGA-PLH-PEG copolymer was coupled to CDA using EDC/NHS chemistry and purified by precipitation and drying in vacuo [14]. After the conjugation reaction, the CDA-AMB-NPs were purified by centrifugation at 14,000 rpm × 30 min and redispersed in phosphate-buffered solution. Finally, CDA-AMB-NPs were lyophilized for 48 h for further use. As a model control molecule, coumarin 6 can be entrapped in PLGA-PLH-PEG NPs for qualitative and quantitative analyses of cellular uptake by candidiasis cells. CDA-coumarin 6-NPs were fabricated in a similar manner.

As a fungal infection usually generates weak localized acidity as far as pH = 6.8 [8–10], the particle size and zeta potential of the PLGA-PLH-PEG NPs in 6.8 and 7.35 pH-adjusted PBS were determined by using a Zeta PALS dynamic light scattering detector (15-mW laser, incident beam 676 nm, Brookhaven Instrument Corporation). PLGA-PLH-PEG NPs were resuspended at 2.5 mg/mL in a 1 % *w*/*v* uranyl acetate solution (pH ~5.5), deposited onto carbon-supported copper TEM grids and dried, and then detected by a JEOL 200 CX TEM (MIT CMSE). And the content of AMB loaded in the micelle was quantified by reverse-phase HPLC. Drug release was conducted by suspending NPs in 4 mL of pH-adjusted PBS, and the drug release rate was measured by spectrophotometry. The released drug was quantified at each time point in triplicate. Drug delivery efficiency in vitro was also detected. 1 × 10^5^*C. albicans* cells were incubated with 10 mL of CDA-coumarin 6-NP solution (equivalent to 1.0 μg/mL coumarin 6) for 12 h at different pH (pH = 6.8 and pH = 7.35) and finally subjected to flow cytometry analysis to determine the intracellular drug delivery efficiency of CDA-drug-NPs.

### Biological Effect of CDA-AMB-NPs

#### Hemolytic Activity

The hemolytic activity of CDA-AMB-NPs was evaluated using human red blood cells (RBCs) [15]. Erythrocytes were washed twice with saline solution and then suspended in saline at 2 % of hematocrit. Different concentrations of AMB solution and AMB-incorporated PLGA-PLH-PEG micelle solution were incubated with properly diluted RBCs at 37 °C for 1 h. After centrifugation at 800 *g* for 15 min, the supernatant was analyzed for hemoglobin at 545 nm using a UV spectrophotometer. The percentage of hemolysis was determined as follows: Hemolysis (%) = [(ODs − OD_min_)/(OD_max_ − OD_min_)] × 100, where ODs is the absorbance of the supernatant from samples incubated with the drugs. Diluted RBCs incubated with PBS and with distilled water were used as minimal (OD_min_) and maximal (OD_max_) hemolytic controls, respectively [16].

#### Determination of Cytotoxicity on HKC

Immortalized human renal tubular epithelial cells (HKC) were cultured at 37 °C with humidified atmosphere of 5 % CO_2_ in DMEM/F12 supplemented with 10 % fetal bovine serum (FBS) and 2 ng/mL mouse epidermal growth factor and were seeded into 96-well culture plates (Corning, Schiphol-Rijk, The Netherlands) for 24 h. Subconfluent cells (culture surface covering nearly 70 %) were then exposed to various AMB or AMB formulation concentrations (1.0–10.0 μM). As kidney injury molecule-1 (KIM-1) and type I transmembrane glycoprotein neutrophil gelatinase-associated lipocalin (NGAL) have been reported to correlate with proximal tubular injury [17, 18], the cytotoxicity was measured periodically by measuring KIM-1 and NGAL using immunofluorescence, WB, and RT-PCR methods to determine early damage caused by exposure to AMB and AMB formulations. The prime set for NGAL was forward, 5′-TCA CCT CCG TCC TGT TTA G-3′ and reverse, 5′-CTC CTT GGT TCT CCC GTA-3′, and that for KIM-1 was forward, 5′-ACTCCTGCAGACTGGAATGG-3′ and reverse, 5′-CAAAGCTCAGAGAGCCCATC-3′. For normalization, the expression of β-actin was examined: forward, 5′-TGC TTC TAG GCG GAC TAT GA-3′ and reverse, 5′-CTC GGC CAC ATT GTG AAC TT-3′ [19].

#### In Vivo Toxicity Analysis

The toxicities of AMB and AMB formulations were assessed with BALB/cA mice (7–9 weeks old). The Committee of Laboratory Animal Welfare and Ethics, Anhui University of Science and Technology has approved all of the animal studies. Five healthy mice were chosen for each route of administration. AMB or AMB formulation was delivered through an intravenous (2.0 mg/kg/day for 3 days, pH = 7.4) route. All animals were monitored through 1 week to determine mortality. Biochemical analyses of blood urea nitrogen (BUN) and creatinine (Cr) levels were determined to assess the nephrotoxicity of CDA-AMB-NPs [16]. And major organs were used for light microscopic evaluation with hematoxylin and eosin (HE) or IHC to evaluate cytological and histological alterations including injury, degeneration, apoptosis, or even necrosis.

#### In Vitro Antimicrobiological Analysis

Susceptibility test for *C. albicans* to different drugs was performed by the agar-based E-test method (bioMérieux, Sweden) with 8.4 g/L RPMI 1640 (RPMI, Sigma Chemical Co., St. Louis, MO, USA), 1.5 % agar, and 1.5 % glucose, poured in 110-mm-diameter plates. The plates were inoculated by dipping a sterile swab into the inoculum suspension adjusted to the turbidity of a 0.5 McFarland standard (10^6^ cells/mL) and streaking it across the surface of the agar in four directions. The plates were dried at ambient temperature for 20 min before applying the E-test strips. The minimum inhibition concentration (MIC) endpoints were determined after 24 h of incubation at 35 °C. The MIC was read for different amphotericin B deforms, and null NPs were used as a blank [20]. Experiments were performed in duplicate.

#### Detection of Apoptosis Markers in C. albicans

*C. albicans* spores (1 × 10^5^ cells/mL) were inoculated in RPMI 1640 medium at 37 °C for 3 h with different antifungal agents (free AMB, AMB-NPs, CDA-AMB-NPs, 10.0 μg/mL) and were incubated for 24 h at 36 °C with shaking (150 rpm). After incubation for 12 h, *C. albicans* cells were harvested and then digested with a lysing enzyme mixture (2.0 U of lyticase, 2.5 U of chitinase, 2.0 U of chitosanase, and 15 mg/mL of lysing enzyme [Sigma]) for 10 h at 35 °C. And the presence of apoptotic markers was analyzed with annexin V-FITC/PI stains. Then, the apoptotic and necrotic features in the drug-treated *C. albicans* cells were evaluated using fluorescence microscopy and flow cytometry instrument detection, respectively.

### In Vivo Antifungal Activity

The in vivo therapeutic efficacy of the prepared nanoparticles was tested by a described method [21]. Sixty 20–22-g BALB/c mice (6–8 weeks old) were infected by inhalation with a volume of 20 μL of PBS containing 5.0 × 10^5^ contagious *C. albicans* cells. After 3 days, all infected mice were randomly divided into four groups. All groups received the drug intravenously as follows: Group A received PBS (10.0 mg/kg/day for 4 days, pH = 7.35); group B received free AMB (10.0 mg/kg/day for 4 days); group C received AMB-NPs (equivalent AMB 10.0 mg/kg/day for 4 days); group D received CDA-AMB-NPs (equivalent AMB 10.0 mg/kg/day for 4 days).

After 2 weeks, the survived mice were anesthetized and sacrificed. Then, the histopathologic changes and fungal colony-forming units (CFU) in the liver, kidney, heart, and right lung were determined.

### Statistical Analysis

Qualitative data was expressed as means ± SEM. Differences between groups were assessed using one-way ANOVA. Differences between paired groups were analysed by Student’s unpaired *t* test. A significance level of *p* < 0.05 was used for all comparisons.

## Discussion

### Polymer Characterization: Morphology, Size, Zeta Potential, Encapsulation Efficiency, and Release

**Fig. 2:**
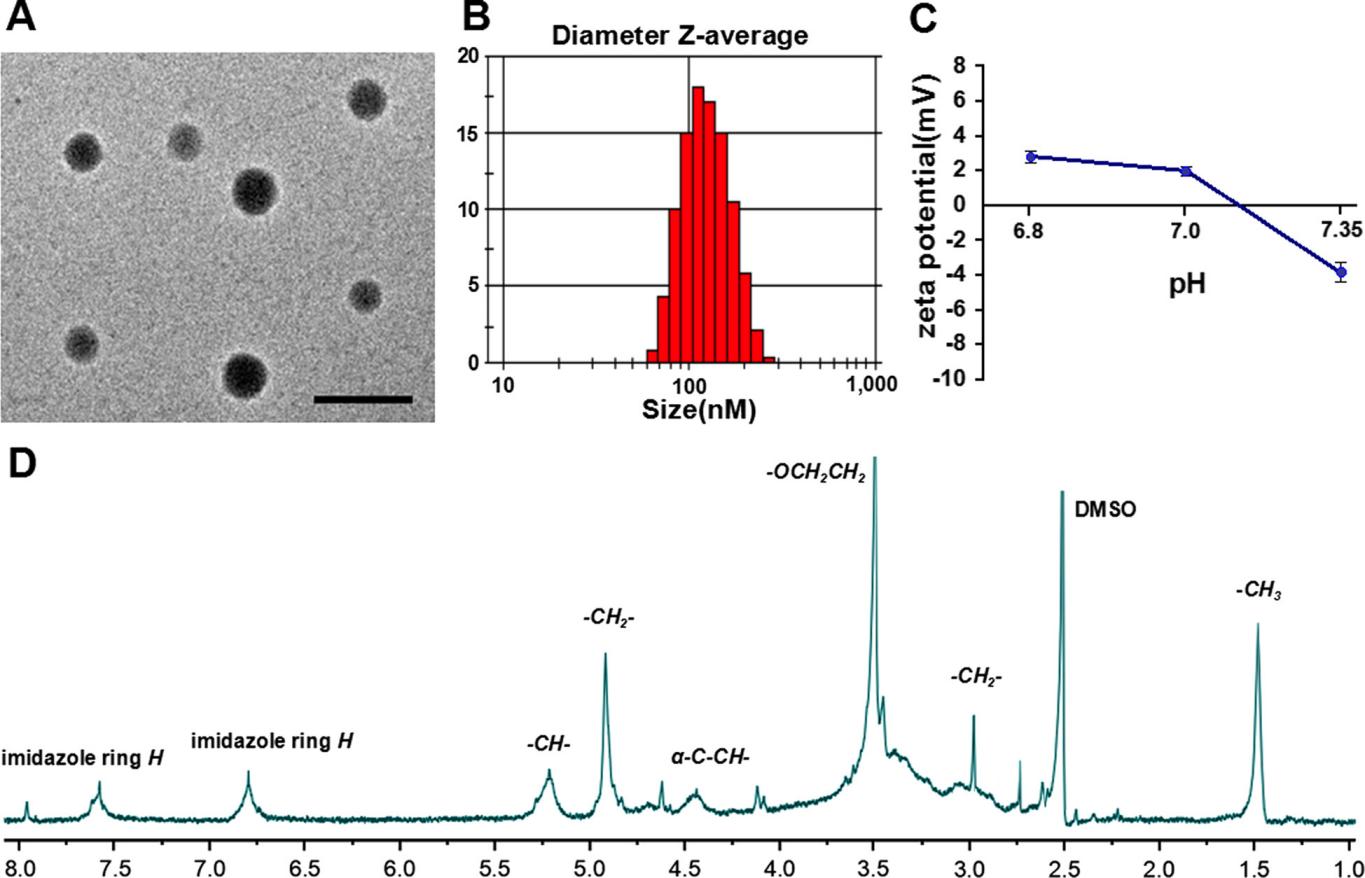
The physical properties of the CDA-AMB-NPs. **a** TEM image shows that the CDA-AMB-NPs were spherical with an average size of 125 nm. **b** Size distribution of the PLGA-PLH-PEG copolymers detected by DLS. **c** Zeta potential vs pH demonstrates notable switching from anionic to cationic with decreases in pH in PLGA-PLH-PEG. **d** ^1^H-NMR spectrum of the PLGA-PLH-PEG copolymer

The physical properties of the CDA-AMB-NPs are displayed in Fig. 2 and Table 1. Size distributions of the NPs analyzed by dynamic light scattering (DLS) were 121.1 ± 3.9 nm, and 121 nm of mean diameter was in the excellent size range for accumulating readily in tumor vasculature due to enhanced retention and permeation effects [20, 21]. The ^1^H-NMR spectrum demonstrates peaks corresponding to the PLGA-PLH-PEG triblock copolymers. Proton shifts in DMSO-d_6_ (ppm): 1.5 b (-C*H*_3_ of LA unit, PLGA), 3.0 b (-C*H*_2_-PLH), 3.5 s (OC*H*_2_C*H*_2_, PEG), 4.4 b (α-carbon-C*H*-, PLH), 4.9 b (-C*H*_2_-, GA unit, PLGA), 5.2 b (-C*H*-LA unit, PLGA), 6.8 b (imidazole ring *H*, PLH), 7.6 b (imidazole ring *H*, PLH).

**Table 1:** Characterization of AMB-loaded nanoparticles (*n* = 3)

Polymer	Particle size (nm)	PDI	EE (%)
PLGA-PLH-PEG(NPs)	109.2 ± 4.1	0.238	–
CDA-AMB-NPs	121.1 ± 3.5	0.215	81.51

*PDI* polydispersity index, *EE* entrapment efficiency

To evaluate the zeta potential (ZP; mV) property which was key to tailoring NP-fungal interactions, CDA-AMB-NPs were resuspended in PBS with appropriate pH (6.8, 7.0, and 7.35). The results showed that the CDA-AMB-NPs switched their surface charge from a negative zeta potential at pH 7.35 (*ζ* = −3.82 ± 0.53 mV, *N* = 3) to a positive one at pH 6.8 (*ζ* = 2.81 ± 0.34 mV, *N* = 3) (Fig. 2c). This change is due to the increasing presence of positive charges from the imidazole group of the PLH with reductions in pH [22]. Polydispersion (PDI), particle size (nm), and EE (%) parameters are displayed in Table 1.

### Intracellular Drug Delivery and Drug Release Efficiency Analysis

**Fig. 3:**
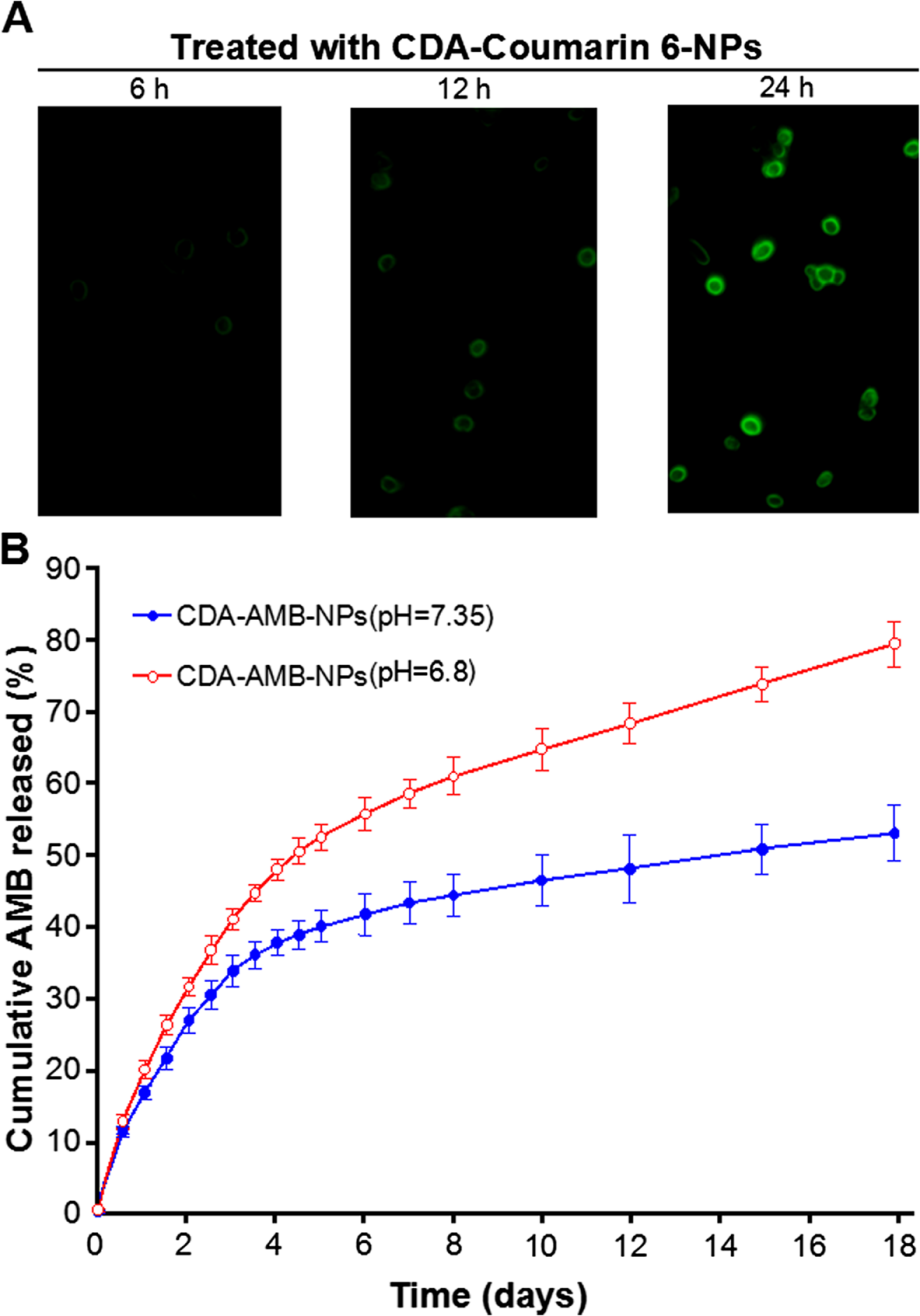
Drug delivery and release efficiency analysis. **a** Fluorescence microscope images (×400) of *C. albicans* cells after being treated with CDA-coumarin 6-NPs. **b** Cumulative release of AMB from CDA-AMB-NPs at 37 °C in pH 6.8 and pH 7.35 in vitro (mean ± SEM, *n* = 5)

Green fluorescence (coumarin 6) as the marker for localization and the uptake and distribution of CDA-coumarin 6-NPs were detected by flow cytometry. The flow cytometry analysis of *C. albicans* cells was carried out 6, 12, and 24 h after the incubation with CDA-coumarin 6-NPs. Figure 3a shows that after 12 h of incubation with CDA-coumarin 6-NPs, the green fluorescence of CDA-coumarin 6-NPs was visible in both the cytoplasm and cellular walls. Moreover, the fluorescence intensity of pH 7.35 was lower than that of pH 6.8.

The in vitro drug release profiles of the freshly prepared CDA-AMB-NPs in PBS (containing 0.1 % *w*/*v* Tween 80) in the first 10 days are displayed in Fig. 3b. Tween 80 was applied to improve the solubility of AMB in the PBS and to avoid the adhesion of AMB onto the tube wall [21, 22]. The AMB release from the CDA-AMB-NPs displayed an initial burst of 58.3 % in the first 6 days at pH = 6.8, which was much higher than that at pH = 7.35. A second slow-release phase sustained for up to 18 days, which was predominantly attributed to the diffusion of the drug. After 18 days, the accumulative AMB release of nanoparticles reached to 81.4 % at pH = 6.8, which indicates that the PLGA-PLH-PEG copolymer was capable of displaying sound drug release at pH = 6.8 (Fig. 3b).

### In Vitro Antifungal Activity

To evaluate the antifungal activity of the AMB-loaded nanoparticles, the minimum inhibitory concentration (MIC) of free AMB, AMB-NPs, and CDA-AMB-NPs at pH 7.35 or pH 6.8 was tested with *C. albicans*. As shown in Table 2, the MIC of AMB-NPs was similar to that of free AMB at pH 7.35 or pH 6.8, which indicates that CDA-AMB-NPs have an antifungal potential similar to that of free AMB.

**Table 2:** Effect of free AMB and AMB-loaded NPs against C. albicans

	MIC (μg/mL, pH = 7.35)	MIC (μg/mL, pH = 6.8)
Free AMB	1.0 ± 0.1	1.0 ± 0.2
AMB-NPs	10.0 ± 0.2	11.0 ± 0.2
CDA-AMB-NPs	11.0 ± 0.2	11.0 ± 0.2

*Abbreviations*: *AMB* amphotericin B, *MIC* minimum inhibitory concentration, *AMB-NPs* 1.0 μg/mL equivalent free AMB 0.1 μg/mL, *CDA-AMB-NPs* 1.2 μg/mL equivalent free AMB 0.1 μg/mL

**Fig. 4:**
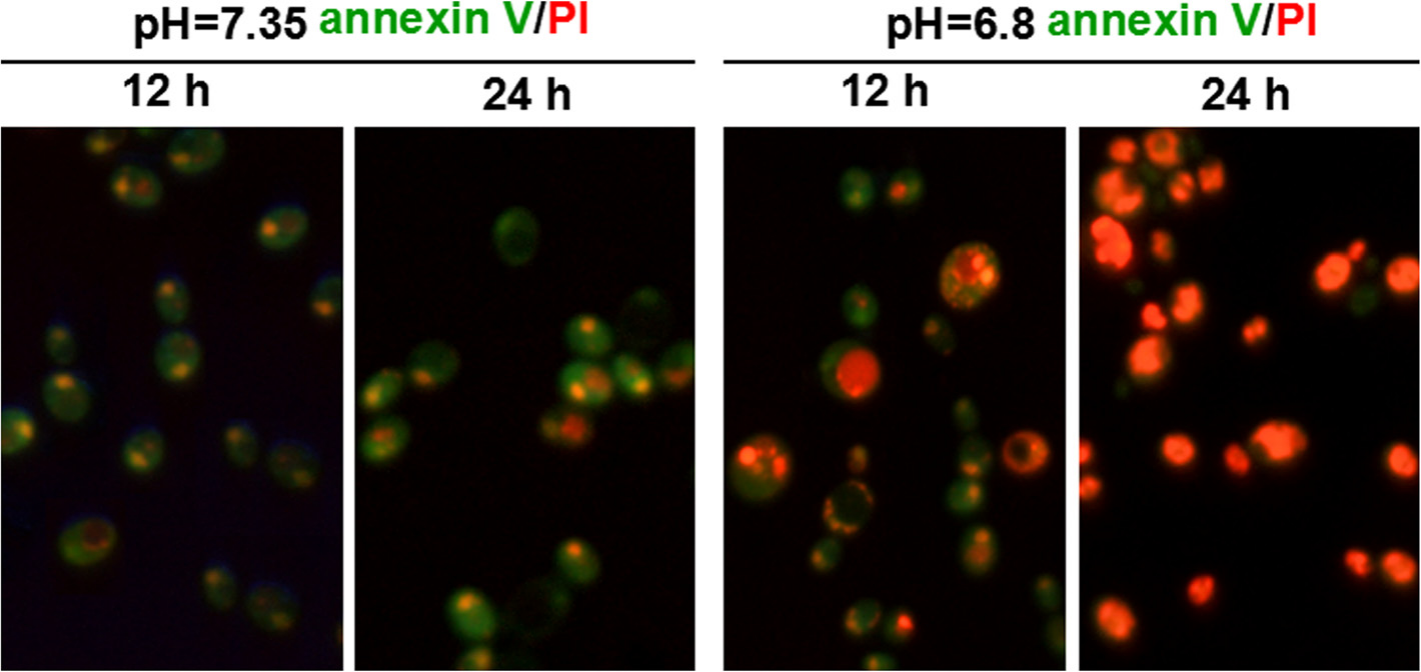
Representative photomicrographs of CDA-AMB-NP-treated *C. albicans* cells. Effects of 12- and 24-h treatment with CDA-AMB-NPs on the activity of *C. albicans* as confirmed using annexin V/PI at pH = 7.35 and pH = 6.8, respectively. Annexin V/PI stains show the presence of necrotic cells (*red fluorescence*) and apoptotic cells (*green fluorescence*). The experiments were performed in triplicate and repeated three times

To investigate whether the drug can effectively induce apoptosis and necrosis of *C. albicans* cells, the apoptotic and necrotic features in CDA-AMB-NP-treated *C. albicans* cells were evaluated using annexin V-FITC and PI double-staining method. Apoptotic cells were stained with annexin V-FITC, whereas necrotic cells were stained with PI accumulated in the nuclei via membrane permeabilization [21]. Following CDA-AMB-NP (6.0 μg/mL, equivalent AMB 0.5 μg/mL) exposure at 37 °C for 24 h at pH = 7.35, about 60 % of the cells were stained with annexin V-FITC, while only 30 % of the cells were stained with PI. When treated with CDA-AMB-NP at pH = 6.8 for the same time, almost all of the cells were stained with annexin V-FITC, and about 90 % of the cells were PI positive as shown in Fig. 4. These results suggest that CDA-AMB-NPs can effectively induce apoptosis and necrosis in *C. albicans* cells at pH = 6.8, mainly attributed to the increased targetability mediated by strong multivalent electrostatic attraction and antibody-antigen binding specificity and affinity effect as well as an improved drug release efficiency (Fig. 3b) which produces a relatively increased drug concentration in localized acidity.

### In Vitro Hemolytic Effects of CDA**-**AMB**-**NPs

In order to determine the effect of CDA-AMB-NPs on hemolytic activity against human erythrocytes, the RBC lysis induced by CDA-AMB-NPs was compared with that induced by free AMB. The extent of hemolysis induced after incubation of RBCs with different formulations of AMB is depicted in Table 3. Contrary to the potent hemolytic activity of free AMB, the tested concentrations of CDA-AMB-NPs exhibited less hemolytic activity toward human erythrocytes (Table 3). Incubation with 5.0 μg/mL of free AMB led to about 20 % RBC lysis after 24 h; however, when free AMB concentration rose up to 20.0 μg/mL, the hemolytic ability significantly increased to 70 %. On the contrary, the RBC lysis in the presence of CDA-AMB-NPs (equivalent to 5.0 and 20.0 μg/mL AMB) was significantly lower compared with that in the presence of the corresponding concentration of free AMB (Table 3). The data indicates the protective role of NP formulations in preventing the hemolytic effects of AMB.

**Table 3:** Hemolytic activity of CDA-AMB-NPs and free AMB against RBCs

Compound	Hemolysis (%)							
	160	80	40	20	10	5.0	2.5	1.25
Free AMB	100	100	100	75.0	35.0	25.0	5.0	0.0
CDA-AMB-NPs	20.0	5.0	1.0	0.0	0.0	0.0	0.0	0.0

### Cytotoxicity on HKC

**Fig. 5:**
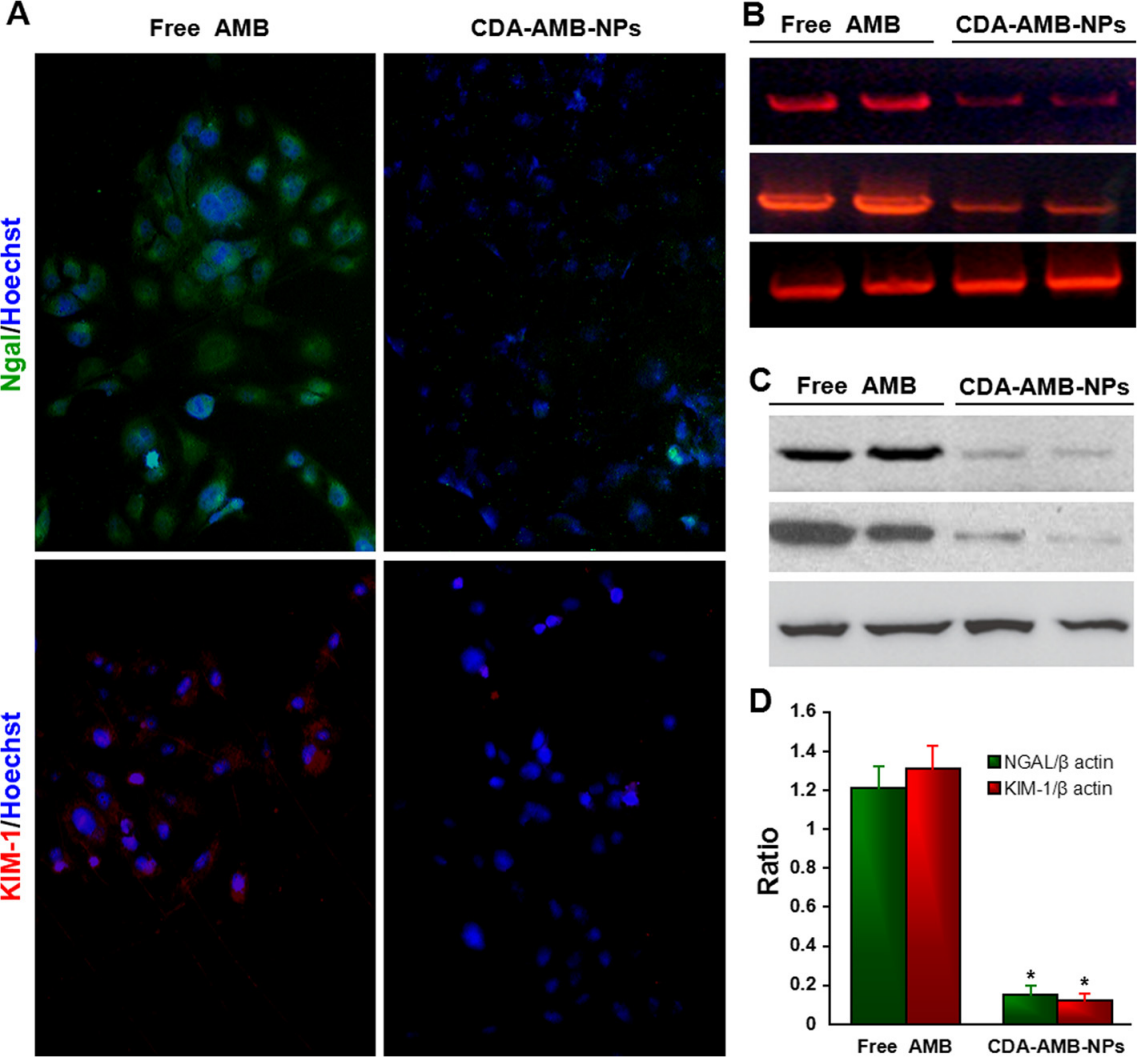
AMB deformations induce HKC damage. Immunofluorescence, RT-PCR, and Western blot were performed to evaluate the cytotoxicity on HKC cells treated with free AMB and CDA-AMB-NPs at 10.0 μM for 48 h. **a** Immunofluorescence staining with anti-KIM-1 and NGAL antibody. KIM-1 was stained with red and NGAL was stained with green. **b** KIM-1 and NGAL mRNA levels were analyzed by RT-PCR. **c**, **d** KIM-1 and NGAL protein levels were analyzed by Western blot. Data are shown as mean ± S.E.M. *p < 0.01, compared with the free AMB group

The in vitro cytotoxicity of CDA-AMB-NPs on the immortalized human renal tubular epithelial cell line (HKC) was evaluated. Free AMB was chosen as reference. The range of concentrations of AMB (1.0, 2.5, 5.0, and 10.0 μM) was selected because it corresponds to plasma levels of the drug achievable in humans [23]. As shown in Fig. 5, HKC cells treated with free AMB or CDA-AMB-NPs (the drug concentration is lower than 2.5 μM) for 48 h, fluorescence almost could not be detected in the two groups. When AMB was increased to 5.0 μM, both red and green fluorescence were detected in HKC, which indicates that HKC expressed KIM-1 and NGAL. HKC cells treated with free AMB (10.0 μM) showed a much stronger intensity of red and green fluorescence. Moreover, mRNA (RT-PCR, Fig. 5b) and protein levels (Western blot, Fig. 5c) also indicate that free AMB causes obvious HKC damage; however, when the concentration of CDA-AMB-NPs was 10.0 μM, the damage on HKC was not obvious (Fig. 5b–d). These results suggest that CDA-AMB-NPs can efficiently reduce the free AMB drug toxicity on HKC cells that originate from the kidney in vitro.

### In Vivo Nephrotoxicity Study

**Fig. 6:**
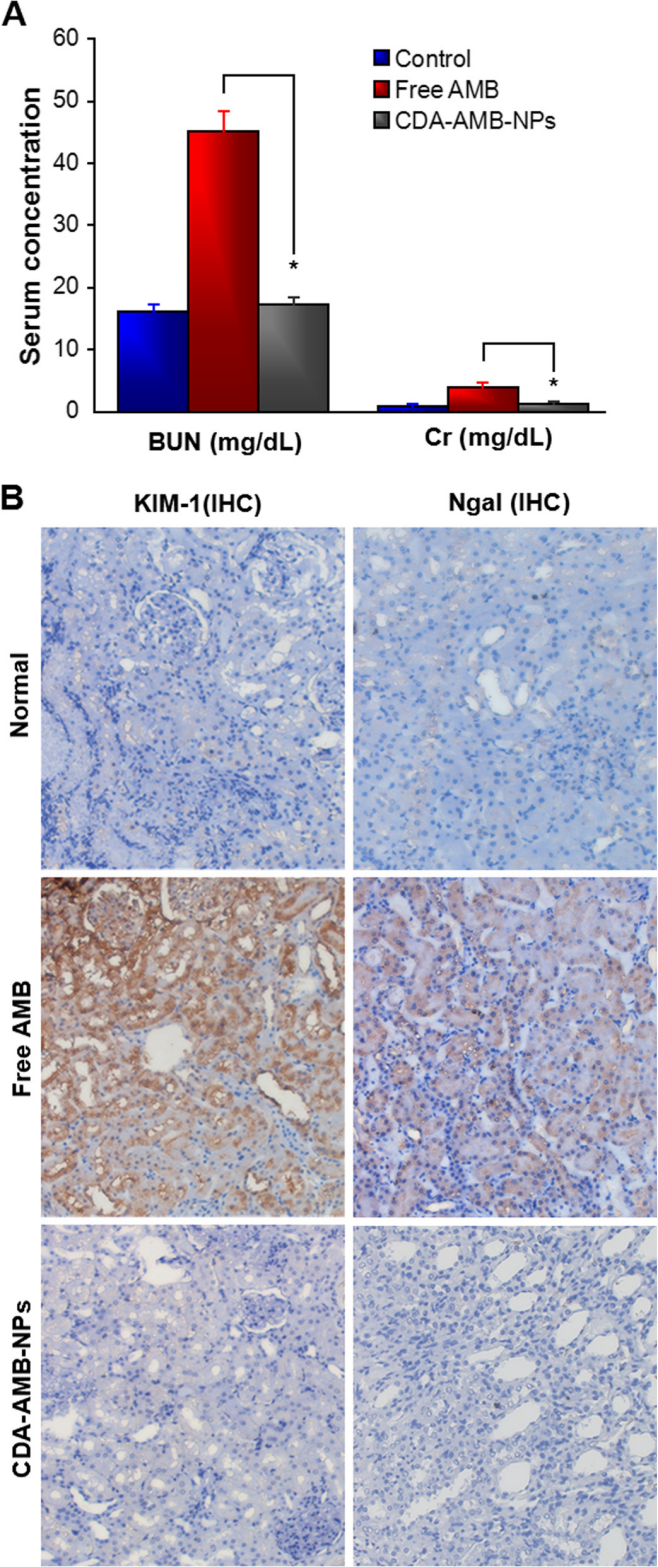
Nephrotoxicity assessment after treatment of different AMB formulations. **a** Blood urea nitrogen (*BUN*) and plasma creatinine (*Cr*) in mice. *p < 0.01, vs free AMB; data presented as mean ± SEM. *n* = 3. **b** Normal mice showed no abnormality and without KIM-1 or NGAL expression. AMB treatment showed high level of expression of KIM-1 and NGAL, but CDA-AMB-NP treatment revealed very low level of expression of KIM-1 and NGAL

The nephrotoxicity of AMB has been manifested by renal insufficiency due to vascular and glomerular disease besides the abnormality in tubular function. As BUN and Cr are important biochemical markers for clinical renal dysfunction, acute AMB-induced nephrotoxicity is characterized by increased BUN and Cr levels. When the mice were administered intravenously with free AMB once a day and for 3 days in total, there would be a significant (*p < 0.01) increase in BUN and Cr levels as compared to normal mice. But CDA-AMB-NPs led to only slightly higher BUN and Cr levels than those of normal mice (*p* > 0.05), indicating lower nephrotoxicity by CDA-AMB-NP compared with free AMB (Fig. 6a). CDA-AMB-NPs also revealed minimal vascular and glomerular damages on the kidney examined by histopathological and immunohistochemical analysis (Fig. 6b).

### Therapeutic Efficacy of CDA**-**AMB**-**NPs

**Fig. 7:**
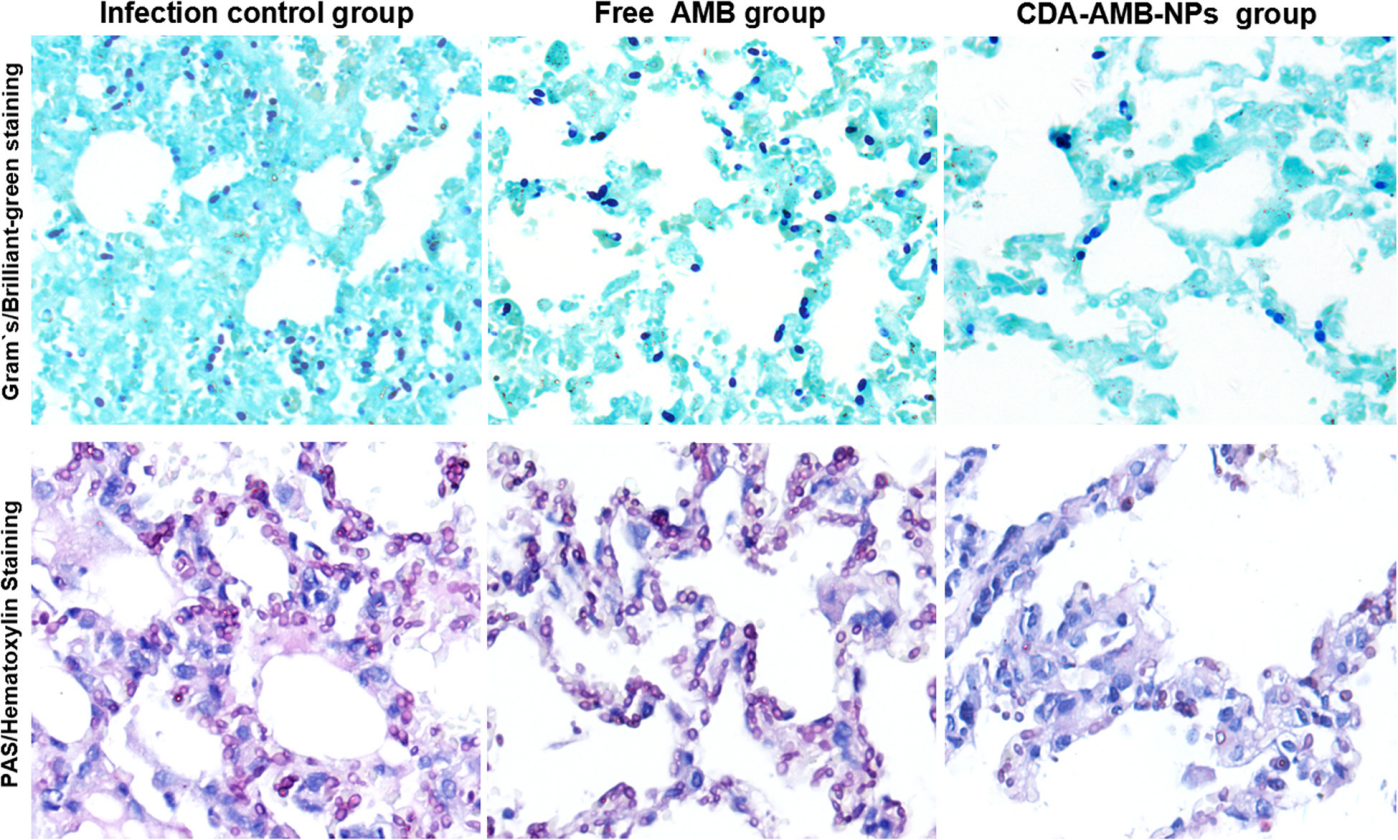
HE analysis. CDA-AMB-NPs and free AMB exhibited different histological patterns of *C. albicans* invasive pneumonia. Representative microscopic images of lung histology from groups of five infected mice following 10 days of intravenous therapy with either CDA-AMB-NPs or AMB at 2.0 mg/kg/day are shown (24 mg of AMB-NPs is equivalent to 2.0 mg of AMB) (×200)

The treatment of *C. albicans*-infected mice with CDA-AMB-NPs compared with controlled animals showed significant reduction in CFU values in evaluated organs (lung, spleen, kidney, and liver), especially in the kidney and spleen. But the therapeutic effect of free AMB was significantly weaker than that of CDA-AMB-NPs (Table 4). Compared with free AMB and PBS treatment, CDA-AMB-NPs effectively eliminated fungi in the kidney, liver, and spleen. And the fungal load in the lungs also significantly reduced (Table 4 and Fig. 7).

**Table 4:** The ratio of survived mice and CFU in different organs of infected mice

	Log CFU gram tissue (*n* = 3)				Survival ratio^a^ (%, *n* = 20)
	Lung	Spleen	Kidney	Liver	
Control (untreated)	4.35 ± 0.27	3.08 ± 0.25	4.78 ± 0.33	3.41 ± 0.34	0.0
PBS	4.29 ± 0.35	3.14 ± 0.21	4.75 ± 0.29	3.34 ± 0.33	0.0
Free AMB	2.51 ± 0.11*	1.12 ± 0.09*	1.21 ± 0.26*	1.87 ± 0.25*	55.0
CDA-AMB-NPs	1.01 ± 0.06*^#^	Nil**	Nil**	Nil**	95.0

Values are expressed as mean ± SEM from four separate experiments. *t* test values (two-tailed) are significant

*p < 0.05, **p < 0.01 vs control; #*p* < 0.01 vs the free AMB group

^a^The survival rate 25 days post infection (*n* = 20)

In in vivo studies, the lesions demonstrated diffuse exudative change accompanied by necrosis and granulomatous formation in the control group. Many hyphae could be found in the lesions. The same pathological changes also existed in the free AMB treatment group. But the therapeutic efficacy of CDA-AMB-NPs was detected in murine candida pneumonia. CDA-AMB-NPs significantly increased the survival time and were more effective in reducing the CFU counts in the lungs than free AMB (Fig. 7).

AMB has been reported to cause hemolysis and cytotoxicity [1, 24]. In order to reduce the toxicity of AMB, we used a nanomaterial which has a slow-release effect to wrap the drug. The result presented that CDA-AMB-NPs increased drug targeting, delayed drug release, prolonged plasma circulation, altered the physical properties of the drug, and reduced hemolytic toxicity (Table 3). The reduction of hemolysis of the free AMB loaded in PLGA-PLH-PEG may be due to the sustained targeting release of the drug in monomeric mode, which reduces the concentration of AMB in the blood, compared with free AMB [25]. CDA-AMB-NPs were also found to be significantly less nephrotoxic, as CDA-AMB-NPs significantly reduced the expression levels of KIM-1 and NGAL which mark the damage of the kidney cells [26] and the BUN and Cr levels in serum. These data demonstrated that CDA-AMB-NPs have therapeutic superiority to free AMB.

To investigate the CDA-AMB-NPs’ targetability, we incubated CDA-coumarin 6-NPs and *C. albicans* together. The results showed that the green fluorescence on the fungal cell wall increased over time (Fig. 3a). In acidic conditions, the release speed of AMB from the nanodrug is more efficient, as CDA-AMB-NPs can more dramatically induce *C. albicans* apoptosis and necrosis at pH 6.8 than free AMB (Fig. 4).

At the same time, the treatment of experimental infection mice with CDA-AMB-NPs resulted in 95 % survival in contrast to 0 % survival in the control group and only 55 % survival in the free AMB group (Table 4). What is more, CDA-AMB-NP treatment also significantly reduced the quantity of pathogens in various organs of the infected mice. And the living bacterium could even be completely eliminated in parts of tissues and organs (Fig. 7 and Table 4). For mice fungal infection of the lungs, both PAS and target blue staining results showed that after the treatment of CDA-AMB-NPs, the quantity of fungal spores in the infected lungs of mice significantly reduced, the alveolar structure returned to normal, and the edema subsided. These results showed that CDA-AMB-NPs can effectively kill fungus, improve lung inflammation and seepage, and promote lung tissue lesion to return to normal physiological status and function. All these efficacy data demonstrated a therapeutic superiority of CDA-AMB-NPs over free AMB.

## Conclusion

The developed CDA-AMB-NPs gave appropriate particle size, extended release, and zeta potential. Compared with AMB, the in vitro*/*in vivo data of the present study against *C. albicans* showed that CDA-AMB-NPs had less toxicity and significantly higher antifungal activity, which indicated that the developed CDA-AMB-NP delivery system may serve as an effective novel low-dose, less side effect antifungal therapeutic system for AMB.

Further experiment on CDA-AMB-NPs including their specific molecular mechanism, targetability, and release mechanism and the efficiency of the loaded drug in fungi-infected lesions will be conducted to reveal more of their characteristics. Moreover, a detailed in vivo study is required before this therapy moves to the bedside. Nevertheless, these results raise interesting possibilities for future studies.
